# A Spectrum of Pulmonary Complications Occurring in End-Stage Renal Disease Patients on Maintenance Hemodialysis

**DOI:** 10.7759/cureus.15426

**Published:** 2021-06-03

**Authors:** Likhita Shaik, Sahith Reddy Thotamgari, Praveen Kowtha, Shaheryar Ranjha, Rutul N Shah, Parneet Kaur, Rashmi Subramani, Renuka R Katta, Abdul mukhtadir Kalaiger, Romil Singh

**Affiliations:** 1 Cardiovascular Disease, Mayo Clinic, Rochester, USA; 2 Research (Cardiovascular Diseases), Mayo Clinic, Rochester, USA; 3 Internal Medicine, Louisiana State University Health Sciences Center, Shreveport, USA; 4 Emergency Department, Ramesh Hospitals, Guntur, IND; 5 Internal Medicine, Ahktar Saeed Medical College, Lahore, PAK; 6 Internal Medicine, M. P. Shah Government Medical College, Jamnagar, IND; 7 Internal Medicine, California Institute of Behavioral Neurosciences & Psychology, Fairfield, USA; 8 Internal Medicine, Department of Health and Family Welfare, Government of Punjab, Chandigarh, IND; 9 Medicine, Sri Guru Ram Das Institute of Medical Sciences and Research, Amritsar, IND; 10 Internal Medicine: Pulmonology, Saveetha Medical College and Hospital, Chennai, IND; 11 Contact Tracer Specialist, Larkin Community Hospital, Florida, USA; 12 Internal Medicine, Mayo Clinic, Rochester, USA; 13 Cardiology, Mayo Clinic, Rochester, USA; 14 Critical Care, Mayo Clinic, Rochester, USA

**Keywords:** acute kidney injury, esrd, pulmonary complications, respiratory tract, kidney disease

## Abstract

Objective

To investigate the trends of end-stage renal disease (ESRD) in patients undergoing maintenance hemodialysis (MHD) and find the correlation with effects on the pulmonary system in such patients.

Methodology

A multicentric prospective study was conducted in the city of Solapur, India. Data were collected from 250 patients through interpersonal interrogation using a questionnaire to capture basic demographic details, the history of ESRD, and relevant respiratory symptoms like breathlessness, cough, fever, etc. related to their disease. Symptoms that are likely associated with the pulmonary system were analyzed and referred to the pulmonology department. Appropriate diagnoses were made using relevant diagnostic tools like X-rays and sputum studies. The association between various disease attributes and pulmonary diagnoses was analyzed using the chi-square (χ2) test, with a p-value of value less than or equal to 0.05 considered statistically significant. Various socio-demographic variables, existing comorbidities, occupation-related risk factors, smoking history, past or current history of any respiratory conditions, the association between the causes of ESRD, time since the first dialysis and sociodemographic factors, and frequency of pulmonary complications were the other covariates in the study.

Results

Our study reports that 31.6% of our patients had significant impairment in their functioning due to respiratory complaints. The prevalence of respiratory complications was 27.2%. Major contributors were pleural effusion (33.8), pneumonia (25), pulmonary edema (20.58), pleuritis (11.76), collapse (8.8), tuberculosis (5.8), fibrosis (4.4), pericardial effusion (4.4), calcification (2.9), and hydrothorax (1.47). We report one case of Urinothorax as a rare cause of hydrothorax in such patients. Overall, our analysis found a significant association between non-reporting of respiratory complaints and acute admissions to the intensive care unit (ICU) with a respiratory cause at p-value 0.0076 with a greater predilection toward the rural populations.

Conclusion

Our study results highlight the prevalence of pulmonary complications in ESRD patients. The occurrence of pulmonary complications, irrespective of the presence of symptoms and a greater association between non-reporting of respiratory symptoms and acute admissions to the ICU, is a hallmark to consider the importance of history and clinical vigilance during patient visits.

## Introduction

End-stage renal disease (ESRD) is defined as an irreversible decline in kidney function, which would otherwise require renal replacement therapy (dialysis or transplantation) [1-2]. It can be implicated from the National Kidney Foundation Kidney Disease Outcomes Quality Initiative classification of chronic kidney disease (CKD) as glomerular filtration rate (GFR) less than 15 ml/min/1.73 m^2^ of body surface area and severely increased albuminuria (>300 mg/g of albumin creatinine ratio or >300 mg of albumin excretion rate in 24 hours) or those requiring renal replacement therapy irrespective of GFR/albuminuria. Such a significant decline in kidney function can lead to a multitude of physiological changes, including fluid retention, cardiovascular complications, respiratory complications, dyselectrolytemia, and anemia [3].

The decreased immune response and alteration in fluid homeostasis in ESRD can present with a plethora of respiratory complications, which include pulmonary edema, fibrinous pleuritis, pulmonary calcification, increased predisposition to tuberculosis, obstructive sleep apnea, and a rarer entity, urinothorax [4]. However, assessment of pulmonary function is not a routine clinical practice in the management of patients with ESRD, and the prevalence and clinical implications of these pulmonary complications in individuals with different etiology of renal impairment are not well-characterized [5]. Moreover, there is less evidence in the literature that could uncover the relation between them.

This study was conducted on patients with various etiologies of ESRD like hypertension, diabetes, autoimmune causes, etc. in different settings (rural vs. urban) to identify both concealed and well-established pulmonary complications. Appropriate interventions were made to address the symptoms and the associated pulmonary complications. Our rationale is to assess (a) the demographic profile of ESRD patients; (b) analyze the factors that affected the disease state; (c) study the pulmonary complications in these patients.

## Materials and methods

Study population

A prospective cohort study was conducted from March 2017 to January 2018 at three hospitals in Solapur, India, among patients with an established ESRD diagnosis. The institutional ethics committee approved the study, and informed consent was obtained from all patients who were willing to participate in the study. The inclusion criteria for the study were adult ESRD patients (age >=18 years) who have been under maintenance hemodialysis (MHD) for at least one year regularly. Patients were secondarily excluded based on age <18 years or have any factors in the history that suggested the presence of confounding variables that could affect the pulmonary system. A total of 250 patients were enrolled in the study who met the inclusion criteria and did not have any relevant and known confounding factors. Selection and information bias was minimized by the random selection of patients by using standardized tools such as questionnaires to obtain information. Data were collected from patients through an interpersonal interrogation through a questionnaire to capture basic demographic details, history of ESRD, and complaints like breathlessness, cough, chest pain, fever, generalized fatigue, weakness, body ache, hemoptysis, and edema. These complaints were analyzed by re-evaluating the history, conducting a physical examination, and referring the patient to a pulmonologist for further evaluation. This evaluation included the use of relevant diagnostic tools like X-rays and sputum studies to diagnose the disease that caused the pertinent symptoms.

Study variables

Potential confounding factors like smoking history, immunodeficiency states, and prior or current respiratory disease were documented. The presence of these factors was used to exclude patients from the study. The history of the disease was gathered from variables like the cause of their ESRD, duration since the diagnosis of ESRD, patterns of hemodialysis, various respiratory complaints (breathlessness, cough, chest pain, fever, generalized fatigue, weakness, body aches, hemoptysis, and edema) and any other pre-existing comorbidities like hypertension, diabetes, interstitial nephropathies, congenital nephropathies, autoimmune diseases, allograft related issues, and renovascular nephropathies. In addition to confounders, limitations were taken into account when interpreting the findings in this study. This study included a diverse patient population of which the most concerning is the location and socioeconomic status. Considering that there may be certain unknown confounders in these groups, our study could not eliminate them. Self-reporting bias was unavoidable due to the nature of this investigation, which analyzed data obtained from the patients' own reporting of their symptoms or complaints. Since the documentation of a positive finding is based on self-reporting of complaints, any concealed disease states could not be accounted for.

Covariates

Sociodemographic variables (e.g., age, gender, socioeconomic status), existing comorbidities (eg, hypertension, diabetes, immunodeficiency states, benign kidney diseases, medication), occupation-related risk factors (e.g., mining, the tobacco industry, pollution-related wort, etc.), smoking history, and past or current history of any respiratory conditions were the covariates in our study. Association between the various attributes of ESRD like the causes of ESRD, time since the first dialysis, sociodemographic factors (e.g., rural, urban living status), and the frequency of pulmonary complications were the other covariates in the study.

Statistical analysis

Categorical data were expressed as proportions while continuous data were expressed as mean values. The distribution of data was expressed in terms of standard deviation (SD). In contingency tables, the significance of the association between the two attributes was analyzed using the chi-square (χ2) test with a p-value of <=0.05 as statistically significant. Data entry and analysis were performed by utilizing the Microsoft Excel version 2104 (Microsoft Corporation, Redmond, WA), JMP version 14.0 (Cary, NC), ​​​​​​and Bluesky Statistics version 7.3 (Chicago, IL) software.

## Results

Out of the 250 patients in our study, 178 (71.2%) were males and 72 (28.8%) were females; 56% resided in rural areas and 44% in urban areas. Patients received hemodialysis either two times per week (85.2%) or three times per week (14.8%). The established causes of their state of chronic kidney disease were categorized as hypertensive (22%) or diabetic (22.8%) nephropathy, analgesic/Interstitial nephritis (9.6%), congenital renal diseases/reflux (8%), glomerulonephritis/autoimmune (6.8%), Renovascular disease (ischemic nephropathy) (4.4%), Polycystic kidney disease (4.4%), Allograft rejection(0.8%) and others (39.6%) [6]. We reported anti-glomerular basement membrane nephritis (1.2%) and immunoglobulin A (IgA) nephritis as autoimmune causes in 1.2% of the total population. 

Seventy-nine (79) out of 250 (31.6%) patients had significant (impaired functioning) respiratory complaints. Breathlessness, at 46 out of 79 (58.2%), was the most common respiratory complaint followed by cough, 18 out of 79 (22.8%). Similarly, the prevalence of other symptoms is listed in Table 1.

**Table 1:** Prevalence of symptoms of patients presenting with respiratory illness

Symptoms	Seen in 79 patients out of 250 (31.6%)
Breathlessness	46 (58.2%)
Cough	18 (22.8%)
Chest pain	6 (7.6%)
Fever	11 (14%)
Generalized (Fatigue, weakness, body aches)	13 (16.4%)
Hemoptysis	4 (4.45%)
Edema	2 (2.53%)

However, irrespective of the complaints, every patient in our study underwent a chest X-ray at the end of the study. The findings conclude that out of 68 patients' significant X-ray findings, 44 did not report respiratory complaints throughout the study. A lesser proportion of patients with more frequent (3 times/week) maintenance hemodialysis (MHD) sessions were diagnosed with pulmonary complications (10 patients, 14.7%) as compared to patients with less frequent MHD sessions (58 patients, 85.3%). The prevalence of pulmonary complications was 27.2% (68) of which pleural effusion was the most common, seen in 23 out of 68 (33.8%) patients. Other pulmonary complications are depicted in Table 2.

**Table 2:** Pulmonary complications

Pulmonary complications	Seen in 68 out of 250 patients (27.2%)
Pleural effusion	23 (33.8%)
Pneumonia	17 (25%)
Pulmonary edema	14 (20.58%)
Pleuritis	8 (11.76%)
Lung collapse	6 (8.8%)
Tuberculosis	4 (5.8%)
Fibrosis	3 (4.4%)
Pericardial effusion	3 (4.4%)
Calcifications	2 (2.9%)
Urinothorax	1 (1.47%)

A highly significant association was found between reporting of complaints and occurrence of pulmonary complications at some point in the study (X2 (1, N = 250) = 14.6309, p = .000131) as shown in Table 3.

**Table 3:** Association between reporting of symptoms and occurrence of complications

Results			
	Complications present	No Complications	Row Totals
Symptoms	34 (21.49) [7.29]	45 (57.51) [2.72]	79
No symptoms	34 (46.51) [3.37]	137 (124.49) [1.26]	171
Column Totals	68	182	250 (Grand Total)

Our study also reported 10 (4%) acute respiratory presentations with ICU admissions during the study span. We observed that out of the 10 acute intensive care unit hospitalizations, three patients never reported any respiratory symptoms during the entire study. The association between not reporting respiratory symptoms and acute admission to the ICU unit for respiratory complications was statistically significant, however, it may not be clinically viable (X2 (1, N = 250) = 7.1064, p =.007681) as demonstrated in Table 4.

**Table 4:** Association between non-reporting of symptoms and ICU admissions ICU: intensive care unit

	ICU admission +	ICU admission -	Marginal Row Totals
No symptoms	3 (6.84) [2.16]	168 (164.16) [0.09]	171
Symptoms present	7 (3.16) [4.67]	72 (75.84) [0.19]	79
Marginal Column Totals	10	240	250 (Grand Total)

## Discussion

ESRD has been a global health issue that not only increases morbidity and mortality but also has a greater impact on quality of life owing to its lifelong dependence on renal replacement therapy or renal transplant. Respiratory illnesses comprise a majority of debilitating complications of ESRD [7]. Many etiologies have been discussed in recent literature, which cause ESRD, including diabetes mellitus, hypertension, glomerulopathies, infection, renal stones, vesicoureteric reflux, and congenital conditions like polycystic kidney disease [4]. The mainstay of our study is to identify the patterns of disease dynamics, treatment dynamics, and the occurrence of pulmonary complications.

The prevalence of pleural effusion as the most common respiratory complication found in our study (9.2%) followed by pneumonia and pulmonary edema was different from the study that reports pulmonary edema as the most common pulmonary complication [7]. Pathogenesis of pleural effusion and pulmonary edema has been explained to be multifactorial, which includes increased hydrostatic pressure due to volume overload and decreased plasma oncotic pressure owing to albumin loss through urine [8-10]. In addition, ventricular systolic dysfunction, which is common in ESRD, can add to the accumulation of fluid in third spaces [11]. Pleural effusion can be unilateral, bilateral, small, or massive based on the etiologies [12]. Urinothorax is an extremely rare complication of ESRD, which is found in 0.4% of the population in our study. It is generally caused by obstructive or malignant pathology of the kidney [8,13]. It occurs when there is communication between renal collecting systems to pleural sacs, leading to urine accumulation in pleural spaces. Many theories have been discussed to know the mechanism of transit of urine, but the exact reason is still not clear. These are generally unilateral and transudative with low pH and a pleural fluid to serum creatinine ratio of more than 1 [14-15]. Inflammation and protein-energy wasting (PEW) are common manifestations found in CKD, which are known to aggravate as the disease progresses to ESRD. Such pathogenesis leads to susceptible infectious and non-infectious pulmonary complications like pneumonia, pleuritis, fibrosis, and calcifications over a long period [16].

In addition to confounders, few limitations were taken into account when interpreting the findings in this study. First, this study included a diverse patient population with regard to age, gender, religion, language, residence, disability, and socioeconomic status, of which the most concerning is the residence and socioeconomic status. Considering that there may be certain unknown confounders in these groups, our study could not eliminate them. Second, the self-reporting bias was unavoidable due to the nature of this investigation. Since the documentation of a positive finding is based on self-reporting of complaints, any concealed disease states could not be accounted for.

This study demonstrates the patterns of pulmonary complications in ESRD patients such that the few most important deductions can be made from these results. The higher reporting of the prevalence rates and the patterns of occurrence of these pulmonary complications in ESRD patients in our study are in agreement with this fact. The higher reporting of symptoms and occurrence of complications in rural populations is in agreement with other studies. This relation between pulmonary complications and the residence of populations is not clear in the literature, which warrants further research. However, this could be attributed to lifestyle habits, socio-economic status, literacy levels, and various other social and personal determinants [17-19].

Moreover, a stronger association found between acute admissions related to pulmonary complications and the non-reporting of any complaints during following visits in our study is a new point of interest in inpatient care. Silent and severe complications like pulmonary embolism, infections, etc., being the prime killers in ESRD populations, must be identified ahead of time through vigilant physical examination and screening tests during primary care and dialysis visits. Such practices can reduce mortality by a significant value [20-23]. The detection of complications without reporting complaints by using screening tools like X-rays is in agreement with this.

In summary, our results show high rates of prevalent pulmonary complications in ESRD patients undergoing dialysis. The patterns of their occurrence highlight that ESRD must be considered a high-risk group for the development of various pulmonary complications that occur irrespective of etiologies, complaints, and the course of the disease. We recommend higher levels of care and vigilance in these patient groups.

## Conclusions

Our study results correlate the prevalence of pulmonary complications in ESRD patients in our study, highlighting pleural effusion to be the most common complication. We recommend the use of more specific diagnostic tools like pleural fluid analysis, cytopathological and microbiological studies, and computed tomography scans to identify extremely rare complications like urinothorax. As per our study, the patterns of X-ray findings, respiratory symptom trends, and acute admissions to the ICU are a hallmark to consider the importance of history and clinical vigilance during patient visits. Efficient tools must be implemented to educate patients about the disease history and its progression to prevent such acute presentations that may lead to poorer and more severe outcomes.

## References

[1] Bush A, Gabriel R (1985). The lungs in uraemia: a review. J R Soc Med.

[2] Prezant DJ (1990). Effect of uremia and its treatment on pulmonary function. Lung.

[3] Mallamaci F, Benedetto FA, Tripepi R (2010). Detection of pulmonary congestion by chest ultrasound in dialysis patients. JACC Cardiovasc Imaging.

[4] Benjamin O, Lappin SL (2021). End-Stage Renal Disease. https://www.ncbi.nlm.nih.gov/books/NBK499861/.

[5] Mukai H, Ming P, Lindholm B, Heimbürger O, Barany P, Stenvinkel P, Qureshi AR (2018). Lung dysfunction and mortality in patients with chronic kidney disease. Kidney Blood Press Res.

[6] Bargman J, Skorecki K (2018). Chapter 280. Chronic kidney disease. Harrison's Principles of Internal Medicine.

[7] Pant P, Baniya S, Jha A (2019). Prevalence of respiratory manifestations in chronic kidney diseases; a descriptive cross-sectional study in a tertiary care hospital of Nepal. JNMA J Nepal Med Assoc.

[8] Pierson DJ (2006). Respiratory considerations in the patient with renal failure. Respir Care.

[9] Wallin CJ, Jacobson SH, Leksell LG (1996). Subclinical pulmonary oedema and intermittent haemodialysis. Nephrol Dial Transplant.

[10] Abdalla ME, AbdElgawad M, Alnahal A (2013). Evaluation of pulmonary function in renal transplant recipients and chronic renal failure patients undergoing maintenance hemodialysis. Egypt J Chest Dis Tuberc.

[11] Zoccali C, Tripepi R, Torino C, Bellantoni M, Tripepi G, Mallamaci F (2013). Lung congestion as a risk factor in end-stage renal disease. Blood Purif.

[12] Yoshii C, Morita S, Tokunaga M (2001). Bilateral massive pleural effusions caused by uremic pleuritis. Intern Med.

[13] Hendriks J, Michielsen D, Van Schil P, Wyndaele JJ (2002). Urinothorax: a rare pleural effusion. Acta Chir Belg.

[14] Salcedo JR (1986). Urinothorax: report of 4 cases and review of the literature. J Urol.

[15] Toubes ME, Lama A, Ferreiro L (2017). Urinothorax: a systematic review. J Thorac Dis.

[16] Mukai H, Ming P, Lindholm B (2018). Restrictive lung disorder is common in patients with kidney failure and associates with protein-energy wasting, inflammation and cardiovascular disease. PLoS One.

[17] Kaze FF, Meto DT, Halle MP, Ngogang J, Kengne AP (2015). Prevalence and determinants of chronic kidney disease in rural and urban Cameroonians: a cross-sectional study. BMC Nephrol.

[18] Maripuri S, Arbogast P, Ikizler TA, Cavanaugh KL (2012). Rural and micropolitan residence and mortality in patients on dialysis. Clin J Am Soc Nephrol.

[19] Eberhardt MS, Pamuk ER (2004). The importance of place of residence: examining health in rural and nonrural areas. Am J Public Health.

[20] Sarnak MJ, Jaber BL (2001). Pulmonary infectious mortality among patients with end-stage renal disease. Chest.

[21] (2021). National Institutes of Health. US Renal Data System. USRDS 1998 annual data report. https://www.usrds.org/annual-data-report/.

[22] Gavelli G, Zompatori M (1997). Thoracic complications in uremic patients and in patients undergoing dialytic treatment: state of the art. Eur Radiol.

[23] (2021). Chronic kidney disease (CKD) clinical presentation. https://emedicine.medscape.com/article/238798-clinical#b3.

